# 4-(4-Chloro­phen­yl)-4-hy­droxy­piperidinium 2-(2-phenyl­eth­yl)benzoate

**DOI:** 10.1107/S1600536811023051

**Published:** 2011-06-18

**Authors:** Hoong-Kun Fun, Madhukar Hemamalini, B. P. Siddaraju, H. S. Yathirajan, B. Narayana

**Affiliations:** a X-ray Crystallography Unit, School of Physics, Universiti Sains Malaysia, 11800 USM, Penang, Malaysia; b Department of Studies in Chemistry, University of Mysore, Manasagangotri, Mysore 570 006, India; c Department of Studies in Chemistry, Mangalore University, Mangalagangotri 574 199, India,

## Abstract

In the title compound, C_11_H_15_ClNO^+^·C_15_H_13_O_2_^−^, the piperidinium ring adopts a chair conformation. In the crystal, cations and anions are connected by inter­molecular O—H⋯O and N—H⋯O hydrogen bonds, forming two-dimensional networks parallel to the *bc* plane. Furthermore, the crystal structure is stabilized by weak C—H⋯π inter­actions.

## Related literature

For related structures, see: Cygler *et al.* (1980 ▶); Cygler & Ahmed, (1984 ▶); Dutkiewicz *et al.* (2010 ▶); Jasinski *et al.* (2009 ▶, 2010 ▶); Tomlin *et al.* (1996 ▶). For the synthesis and biological activity of uncondensed cyclic derivatives of piperidine, see: Vartanyan (1984 ▶). For ring conformations, see: Cremer & Pople (1975 ▶). For the stability of the temperature controller used in the data collection, see: Cosier & Glazer (1986 ▶).
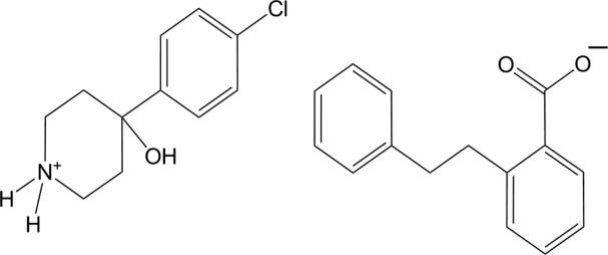


## Experimental

### 

#### Crystal data

C_11_H_15_ClNO^+^·C_15_H_13_O_2_^−^
*M* *_r_* = 437.94
Monoclinic, 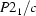

*a* = 13.1016 (2) Å
*b* = 10.2963 (2) Å
*c* = 16.8015 (3) Å
β = 98.234 (1)°
*V* = 2243.12 (7) Å^3^
*Z* = 4
Mo *K*α radiation
μ = 0.20 mm^−1^
*T* = 100 K
0.45 × 0.43 × 0.33 mm

#### Data collection

Bruker SMART APEXII CCD area-detector diffractometer
Absorption correction: multi-scan (*SADABS*; Bruker, 2009 ▶) *T*_min_ = 0.916, *T*_max_ = 0.938
27219 measured reflections
8207 independent reflections
6646 reflections with *I* > 2σ(*I*)
*R*_int_ = 0.025

#### Refinement

*R*[*F* ^2^ > 2σ(*F* ^2^)] = 0.042
*wR*(*F* ^2^) = 0.120
*S* = 1.03
8207 reflections
292 parameters
H atoms treated by a mixture of independent and constrained refinement
Δρ_max_ = 0.44 e Å^−3^
Δρ_min_ = −0.36 e Å^−3^

### 

Data collection: *APEX2* (Bruker, 2009 ▶); cell refinement: *SAINT* (Bruker, 2009 ▶); data reduction: *SAINT*; program(s) used to solve structure: *SHELXTL* (Sheldrick, 2008 ▶); program(s) used to refine structure: *SHELXTL*; molecular graphics: *SHELXTL*; software used to prepare material for publication: *SHELXTL* and *PLATON* (Spek, 2009 ▶).

## Supplementary Material

Crystal structure: contains datablock(s) global, I. DOI: 10.1107/S1600536811023051/rz2607sup1.cif

Structure factors: contains datablock(s) I. DOI: 10.1107/S1600536811023051/rz2607Isup2.hkl

Supplementary material file. DOI: 10.1107/S1600536811023051/rz2607Isup3.cml

Additional supplementary materials:  crystallographic information; 3D view; checkCIF report

## Figures and Tables

**Table 1:** Hydrogen-bond geometry (Å, °) *Cg*2 is the centroid of the C20–C25 ring.

*D*—H⋯*A*	*D*—H	H⋯*A*	*D*⋯*A*	*D*—H⋯*A*
O1—H1*O*1⋯O3	0.890 (18)	1.891 (18)	2.7401 (12)	158.8 (16)
N1—H1*N*1⋯O3^i^	0.954 (16)	1.754 (16)	2.6939 (11)	167.7 (15)
N1—H2*N*1⋯O2^ii^	0.917 (16)	1.818 (16)	2.7223 (11)	168.6 (15)
C8—H8*B*⋯*Cg*2	0.99	2.85	3.6743 (11)	141

Symmetry codes: (i) 
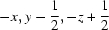
; (ii) 
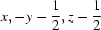
.

**Fig. 1.**
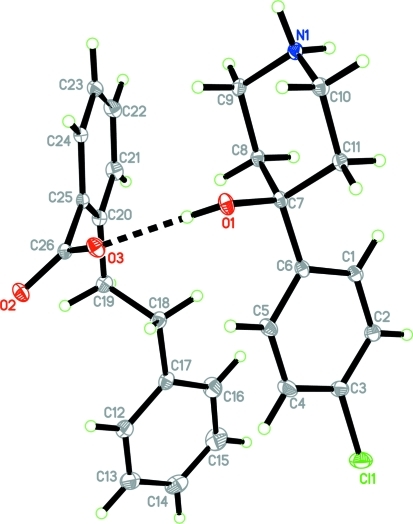
The asymmetric unit of the title compound, showing 50% probability displacement ellipsoids. Intermolecular hydrogen bond is shown as a dashed line.

**Fig. 2.**
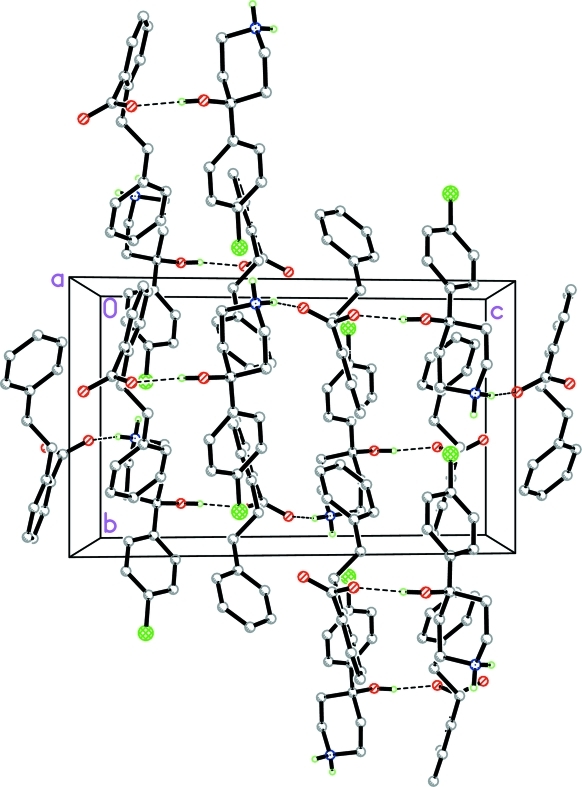
The crystal packing of the title compound. Intermolecular hydrogen bonds are shown as dashed lines. Hydrogen atoms not involved in hydrogen bonding are omitted for clarity.

## supplementary crystallographic information

### Comment

4-(4-Chlorophenyl)-4-hydroxypiperidine is used as an intermediate for the synthesis of pharmaceuticals such as haloperidol (neuroleptic drug used to treat patients with psychotic illnesses, extreme agitation, or Tourette's syndrome) and loperamide, which is a synthetic piperidine derivative, is a drug effective against diarrhea resulting from gastroenteritis or inflammatory bowel disease. A review on the synthesis and biological activity of uncondensed cyclic derivatives of piperidine has been reported (Vartanyan, 1984).

The crystal structures of  1,2,2,4,6,6-hexamethyl-4-piperidinol (Cygler *et al.*, 1980), three isomers of (±)-1,2,3-trimethyl-4-phenyl- 4-piperidinol (Cygler & Ahmed, 1984), 1-(4-nitrophenyl)-4-piperidinol (Tomlin *et al.*, 1996) and 4-[(*E*)-(2,4-difluorophenyl) (hydroxyimino)methyl]piperidinium picrate (Jasinski *et al.*, 2009) have been reported. Also the crystal structures of 4-(4-chlorophenyl) piperidin-4-ol (Dutkiewicz *et al.*, 2010) and 4-(4-chlorophenyl)- 4-hydroxypiperidinium maleate maleic acid solvate (Jasinski *et al.*, 2010) have been reported. In view of the importance of the title compound (I), its crystal structure is reported herein.

The asymmetric unit of (I), (Fig.1), consists of a 4-(4-chlorophenyl)-4- hydroxypiperidinium cation and a 2-(2-phenylethyl)benzoate anion.  The piperidine ring adopts a chair conformation with puckering parameters Q = 0.5678 (10) Å, θ = 1.81 (10)° and φ = 198 (3)° (Cremer & Pople, 1975). In the cation, the dihedral angle between the mean planes of the piperidinium ring  (N1/C7–C11) with the benzene ring (C1–C6) is 88.12 (5)°. In the anion, the dihedral angle between the benzene (C12–C17) ring and the carboxy-subsituted phenyl (C20–C25) ring is 40.72 (5)°. In the crystal structure (Fig. 2), the cations and anions are connected by intermolecular N1—H1N1···O3, N1—H2N1···O3 and O1—H1O1···O3 hydrogen bonds, forming  two-dimensional networks parallel to the *bc* plane. Furthermore, the crystal structure is stabilized by  weak C—H···π interactions, involving the C20–C25 ring.

### Experimental

4-(4-Chlorophenyl)-piperidin-4-ol (2.12 g, 0.01 mol) was dissolved in 10 ml of methanol and 2-(2-phenylethyl)benzoic acid (2.26 g, 0.01 mol) was dissolved in 10 ml of methanol. The solutions were mixed and stirred in a beaker at 333 K for 30 minutes. The mixture was kept aside for three days at room temperature. The formed salt was filtered and dried in vacuum desiccator over phosphorous pentoxide. Crystals suitable for X-ray analysis were obtained by slow evaporation of a N,N-dimethylformamide solution (m. p.: 445–448 K).

### Refinement

Atoms H1O1, H1N1, H2N1 were located in difference Fourier maps and refined freely [N–H = 0.917 (16)–0.954 (16) Å; O–H = 0.890 (18) Å]. The remaining H atoms were positioned geometrically [C–H = 0.95 or 0.99 Å] and were refined using a riding model, with *U*_iso_(H) = 1.2 *U*_eq_(C).

### Crystal data

**Table:**

C_11_H_15_ClNO^+^·C_15_H_13_O_2_^−^	*F*(000) = 928
*M**_r_* = 437.94	*D*_x_ = 1.297 Mg m^−^^3^
Monoclinic, *P*2_1_/*c*	Mo *K*α radiation, λ = 0.71073 Å
Hall symbol: -P 2ybc	Cell parameters from 9967 reflections
*a* = 13.1016 (2) Å	θ = 2.5–32.7°
*b* = 10.2963 (2) Å	µ = 0.20 mm^−^^1^
*c* = 16.8015 (3) Å	*T* = 100 K
β = 98.234 (1)°	Block, colourless
*V* = 2243.12 (7) Å^3^	0.45 × 0.43 × 0.33 mm
*Z* = 4	

### Data collection

**Table:**

Bruker SMART APEXII CCD area-detector diffractometer	8207 independent reflections
Radiation source: fine-focus sealed tube	6646 reflections with *I* > 2σ(*I*)
graphite	*R*_int_ = 0.025
φ and ω scans	θ_max_ = 32.7°, θ_min_ = 1.6°
Absorption correction: multi-scan (*SADABS*; Bruker, 2009)	*h* = −19→19
*T*_min_ = 0.916, *T*_max_ = 0.938	*k* = −15→15
27219 measured reflections	*l* = −25→25

### Refinement

**Table:**

Refinement on *F*^2^	Primary atom site location: structure-invariant direct methods
Least-squares matrix: full	Secondary atom site location: difference Fourier map
*R*[*F*^2^ > 2σ(*F*^2^)] = 0.042	Hydrogen site location: inferred from neighbouring sites
*wR*(*F*^2^) = 0.120	H atoms treated by a mixture of independent and constrained refinement
*S* = 1.03	*w* = 1/[σ^2^(*F*_o_^2^) + (0.0626*P*)^2^ + 0.5497*P*] where *P* = (*F*_o_^2^ + 2*F*_c_^2^)/3
8207 reflections	(Δ/σ)_max_ = 0.001
292 parameters	Δρ_max_ = 0.44 e Å^−^^3^
0 restraints	Δρ_min_ = −0.36 e Å^−^^3^

### Special details

**Table:**

Experimental. The crystal was placed in the cold stream of an Oxford Cryosystems Cobra open-flow nitrogen cryostat (Cosier & Glazer, 1986) operating at 100.0 (1) K.
Geometry. All s.u.'s (except the s.u. in the dihedral angle between two l.s. planes) are estimated using the full covariance matrix. The cell s.u.'s are taken into account individually in the estimation of s.u.'s in distances, angles and torsion angles; correlations between s.u.'s in cell parameters are only used when they are defined by crystal symmetry. An approximate (isotropic) treatment of cell s.u.'s is used for estimating s.u.'s involving l.s. planes.
Refinement. Refinement of F^2^ against ALL reflections. The weighted R-factor wR and goodness of fit S are based on F^2^, conventional R-factors R are based on F, with F set to zero for negative F^2^. The threshold expression of F^2^ > 2σ(F^2^) is used only for calculating R-factors(gt) etc. and is not relevant to the choice of reflections for refinement. R-factors based on F^2^ are statistically about twice as large as those based on F, and R- factors based on ALL data will be even larger.

### Fractional atomic coordinates and isotropic or equivalent isotropic displacement parameters (Å^2^)

**Table:**

	*x*	*y*	*z*	*U*_iso_*/*U*_eq_	
Cl1	0.31506 (2)	0.35787 (3)	0.13139 (2)	0.03391 (8)	
O1	0.00926 (5)	−0.14140 (7)	0.20489 (5)	0.02077 (15)	
N1	0.03580 (6)	−0.41264 (8)	0.08973 (5)	0.01795 (15)	
C1	0.20108 (7)	0.00102 (10)	0.08361 (6)	0.01995 (18)	
H1A	0.2080	−0.0661	0.0460	0.024*	
C2	0.25545 (8)	0.11629 (10)	0.07964 (6)	0.02206 (19)	
H2A	0.2990	0.1280	0.0396	0.026*	
C3	0.24543 (8)	0.21405 (10)	0.13489 (7)	0.02213 (19)	
C4	0.18101 (8)	0.19870 (10)	0.19279 (7)	0.0250 (2)	
H4A	0.1737	0.2665	0.2299	0.030*	
C5	0.12706 (8)	0.08269 (10)	0.19592 (6)	0.02169 (19)	
H5A	0.0830	0.0720	0.2357	0.026*	
C6	0.13625 (7)	−0.01800 (9)	0.14203 (6)	0.01690 (16)	
C7	0.07888 (7)	−0.14634 (9)	0.14699 (6)	0.01646 (16)	
C8	0.15729 (7)	−0.25735 (9)	0.16651 (6)	0.01683 (16)	
H8A	0.2050	−0.2586	0.1259	0.020*	
H8B	0.1985	−0.2414	0.2197	0.020*	
C9	0.10419 (7)	−0.38846 (10)	0.16734 (6)	0.01883 (17)	
H9A	0.1569	−0.4579	0.1765	0.023*	
H9B	0.0627	−0.3910	0.2121	0.023*	
C10	−0.04213 (7)	−0.30767 (10)	0.06881 (6)	0.02007 (18)	
H10A	−0.0903	−0.3059	0.1091	0.024*	
H10B	−0.0825	−0.3260	0.0156	0.024*	
C11	0.01043 (7)	−0.17595 (9)	0.06675 (6)	0.01840 (17)	
H11A	−0.0427	−0.1074	0.0555	0.022*	
H11B	0.0532	−0.1751	0.0227	0.022*	
O2	0.17229 (6)	−0.09424 (8)	0.48194 (4)	0.02185 (15)	
O3	0.06394 (5)	−0.12510 (7)	0.36821 (5)	0.02150 (15)	
C12	0.40341 (9)	0.18559 (11)	0.42461 (7)	0.0259 (2)	
H12A	0.3526	0.1706	0.4586	0.031*	
C13	0.47129 (9)	0.28931 (11)	0.44020 (8)	0.0308 (2)	
H13A	0.4669	0.3443	0.4850	0.037*	
C14	0.54514 (9)	0.31271 (12)	0.39082 (9)	0.0347 (3)	
H14A	0.5915	0.3836	0.4016	0.042*	
C15	0.55121 (10)	0.23302 (15)	0.32602 (10)	0.0405 (3)	
H15A	0.6015	0.2494	0.2918	0.049*	
C16	0.48372 (9)	0.12813 (13)	0.31033 (8)	0.0332 (3)	
H16A	0.4888	0.0732	0.2656	0.040*	
C17	0.40923 (7)	0.10336 (10)	0.35948 (6)	0.02131 (18)	
C18	0.34031 (8)	−0.01409 (10)	0.34599 (7)	0.02296 (19)	
H18A	0.3426	−0.0488	0.2913	0.028*	
H18B	0.2683	0.0115	0.3497	0.028*	
C19	0.37437 (7)	−0.12055 (10)	0.40862 (7)	0.02103 (18)	
H19A	0.4493	−0.1358	0.4107	0.025*	
H19B	0.3623	−0.0897	0.4623	0.025*	
C20	0.31783 (7)	−0.24719 (9)	0.39020 (6)	0.01833 (17)	
C21	0.37025 (8)	−0.35504 (11)	0.36547 (7)	0.0240 (2)	
H21A	0.4418	−0.3476	0.3622	0.029*	
C22	0.32102 (9)	−0.47260 (10)	0.34553 (7)	0.0254 (2)	
H22A	0.3588	−0.5441	0.3291	0.031*	
C23	0.21641 (8)	−0.48510 (10)	0.34974 (6)	0.02224 (19)	
H23A	0.1825	−0.5658	0.3377	0.027*	
C24	0.16174 (8)	−0.37824 (9)	0.37178 (6)	0.01810 (17)	
H24A	0.0897	−0.3858	0.3730	0.022*	
C25	0.21110 (7)	−0.26014 (9)	0.39210 (5)	0.01569 (16)	
C26	0.14577 (7)	−0.15046 (9)	0.41621 (6)	0.01634 (16)	
H1O1	0.0426 (13)	−0.1412 (16)	0.2549 (11)	0.040 (4)*	
H1N1	−0.0007 (12)	−0.4910 (15)	0.0969 (10)	0.032 (4)*	
H2N1	0.0777 (12)	−0.4202 (16)	0.0507 (10)	0.033 (4)*	

### Atomic displacement parameters (Å^2^)

**Table:**

	*U*^11^	*U*^22^	*U*^33^	*U*^12^	*U*^13^	*U*^23^
Cl1	0.04360 (17)	0.02108 (12)	0.04013 (17)	−0.00762 (10)	0.01656 (13)	−0.00582 (10)
O1	0.0165 (3)	0.0312 (4)	0.0156 (3)	−0.0001 (3)	0.0057 (3)	−0.0022 (3)
N1	0.0182 (3)	0.0205 (4)	0.0155 (4)	−0.0027 (3)	0.0033 (3)	0.0009 (3)
C1	0.0230 (4)	0.0201 (4)	0.0178 (4)	0.0008 (3)	0.0066 (3)	−0.0022 (3)
C2	0.0258 (4)	0.0206 (4)	0.0214 (5)	−0.0005 (3)	0.0089 (4)	−0.0008 (3)
C3	0.0239 (4)	0.0183 (4)	0.0246 (5)	0.0001 (3)	0.0050 (4)	−0.0016 (3)
C4	0.0281 (5)	0.0227 (4)	0.0255 (5)	0.0008 (4)	0.0085 (4)	−0.0078 (4)
C5	0.0211 (4)	0.0248 (4)	0.0206 (5)	0.0004 (3)	0.0079 (4)	−0.0059 (4)
C6	0.0155 (4)	0.0201 (4)	0.0151 (4)	0.0015 (3)	0.0022 (3)	−0.0012 (3)
C7	0.0153 (4)	0.0209 (4)	0.0136 (4)	0.0004 (3)	0.0035 (3)	−0.0013 (3)
C8	0.0149 (3)	0.0208 (4)	0.0149 (4)	−0.0007 (3)	0.0026 (3)	0.0006 (3)
C9	0.0188 (4)	0.0227 (4)	0.0148 (4)	−0.0016 (3)	0.0018 (3)	0.0030 (3)
C10	0.0168 (4)	0.0243 (4)	0.0186 (4)	−0.0008 (3)	0.0006 (3)	−0.0005 (3)
C11	0.0183 (4)	0.0208 (4)	0.0157 (4)	0.0011 (3)	0.0010 (3)	−0.0006 (3)
O2	0.0217 (3)	0.0268 (3)	0.0178 (3)	−0.0008 (3)	0.0056 (3)	−0.0061 (3)
O3	0.0197 (3)	0.0251 (3)	0.0195 (3)	0.0051 (3)	0.0021 (3)	−0.0039 (3)
C12	0.0264 (5)	0.0253 (5)	0.0263 (5)	−0.0002 (4)	0.0050 (4)	0.0022 (4)
C13	0.0326 (5)	0.0245 (5)	0.0331 (6)	−0.0002 (4)	−0.0027 (5)	0.0002 (4)
C14	0.0249 (5)	0.0297 (5)	0.0469 (8)	−0.0076 (4)	−0.0040 (5)	0.0058 (5)
C15	0.0284 (5)	0.0478 (7)	0.0478 (8)	−0.0146 (5)	0.0141 (5)	0.0033 (6)
C16	0.0292 (5)	0.0400 (6)	0.0333 (6)	−0.0091 (5)	0.0148 (5)	−0.0026 (5)
C17	0.0174 (4)	0.0240 (4)	0.0225 (5)	−0.0015 (3)	0.0029 (3)	0.0044 (4)
C18	0.0214 (4)	0.0247 (4)	0.0227 (5)	−0.0035 (3)	0.0029 (4)	0.0030 (4)
C19	0.0163 (4)	0.0233 (4)	0.0234 (5)	−0.0012 (3)	0.0025 (3)	0.0023 (4)
C20	0.0163 (4)	0.0209 (4)	0.0178 (4)	0.0021 (3)	0.0026 (3)	0.0017 (3)
C21	0.0191 (4)	0.0285 (5)	0.0245 (5)	0.0070 (4)	0.0040 (4)	−0.0010 (4)
C22	0.0286 (5)	0.0239 (5)	0.0234 (5)	0.0095 (4)	0.0025 (4)	−0.0021 (4)
C23	0.0303 (5)	0.0182 (4)	0.0179 (4)	0.0023 (4)	0.0023 (4)	−0.0008 (3)
C24	0.0210 (4)	0.0191 (4)	0.0144 (4)	−0.0008 (3)	0.0034 (3)	0.0003 (3)
C25	0.0172 (4)	0.0177 (4)	0.0125 (4)	0.0011 (3)	0.0030 (3)	0.0008 (3)
C26	0.0165 (4)	0.0175 (4)	0.0162 (4)	−0.0008 (3)	0.0064 (3)	0.0003 (3)

### Geometric parameters (Å, °)

**Table:**

Cl1—C3	1.7447 (10)	O3—C26	1.2728 (12)
O1—C7	1.4265 (11)	C12—C13	1.3905 (16)
O1—H1O1	0.890 (18)	C12—C17	1.3943 (16)
N1—C9	1.4934 (13)	C12—H12A	0.9500
N1—C10	1.4938 (13)	C13—C14	1.3829 (19)
N1—H1N1	0.954 (16)	C13—H13A	0.9500
N1—H2N1	0.917 (16)	C14—C15	1.375 (2)
C1—C2	1.3908 (14)	C14—H14A	0.9500
C1—C6	1.4008 (13)	C15—C16	1.3967 (18)
C1—H1A	0.9500	C15—H15A	0.9500
C2—C3	1.3881 (14)	C16—C17	1.3893 (15)
C2—H2A	0.9500	C16—H16A	0.9500
C3—C4	1.3852 (15)	C17—C18	1.5068 (14)
C4—C5	1.3928 (15)	C18—C19	1.5406 (15)
C4—H4A	0.9500	C18—H18A	0.9900
C5—C6	1.3929 (13)	C18—H18B	0.9900
C5—H5A	0.9500	C19—C20	1.5097 (14)
C6—C7	1.5284 (13)	C19—H19A	0.9900
C7—C11	1.5390 (13)	C19—H19B	0.9900
C7—C8	1.5404 (13)	C20—C21	1.3996 (13)
C8—C9	1.5196 (13)	C20—C25	1.4096 (12)
C8—H8A	0.9900	C21—C22	1.3898 (16)
C8—H8B	0.9900	C21—H21A	0.9500
C9—H9A	0.9900	C22—C23	1.3885 (16)
C9—H9B	0.9900	C22—H22A	0.9500
C10—C11	1.5237 (14)	C23—C24	1.3916 (13)
C10—H10A	0.9900	C23—H23A	0.9500
C10—H10B	0.9900	C24—C25	1.3966 (13)
C11—H11A	0.9900	C24—H24A	0.9500
C11—H11B	0.9900	C25—C26	1.5071 (12)
O2—C26	1.2513 (12)		
			
C7—O1—H1O1	111.7 (11)	C7—C11—H11B	109.3
C9—N1—C10	112.91 (8)	H11A—C11—H11B	108.0
C9—N1—H1N1	106.3 (10)	C13—C12—C17	120.59 (10)
C10—N1—H1N1	107.7 (9)	C13—C12—H12A	119.7
C9—N1—H2N1	107.0 (10)	C17—C12—H12A	119.7
C10—N1—H2N1	110.6 (10)	C14—C13—C12	120.29 (12)
H1N1—N1—H2N1	112.3 (14)	C14—C13—H13A	119.9
C2—C1—C6	121.27 (9)	C12—C13—H13A	119.9
C2—C1—H1A	119.4	C15—C14—C13	119.73 (11)
C6—C1—H1A	119.4	C15—C14—H14A	120.1
C3—C2—C1	119.24 (9)	C13—C14—H14A	120.1
C3—C2—H2A	120.4	C14—C15—C16	120.27 (12)
C1—C2—H2A	120.4	C14—C15—H15A	119.9
C4—C3—C2	120.83 (9)	C16—C15—H15A	119.9
C4—C3—Cl1	119.64 (8)	C17—C16—C15	120.62 (12)
C2—C3—Cl1	119.53 (8)	C17—C16—H16A	119.7
C3—C4—C5	119.20 (9)	C15—C16—H16A	119.7
C3—C4—H4A	120.4	C16—C17—C12	118.49 (10)
C5—C4—H4A	120.4	C16—C17—C18	120.98 (10)
C4—C5—C6	121.48 (9)	C12—C17—C18	120.43 (9)
C4—C5—H5A	119.3	C17—C18—C19	111.07 (9)
C6—C5—H5A	119.3	C17—C18—H18A	109.4
C5—C6—C1	117.96 (9)	C19—C18—H18A	109.4
C5—C6—C7	121.28 (8)	C17—C18—H18B	109.4
C1—C6—C7	120.75 (8)	C19—C18—H18B	109.4
O1—C7—C6	111.95 (7)	H18A—C18—H18B	108.0
O1—C7—C11	104.79 (7)	C20—C19—C18	112.89 (8)
C6—C7—C11	110.66 (8)	C20—C19—H19A	109.0
O1—C7—C8	110.69 (7)	C18—C19—H19A	109.0
C6—C7—C8	109.57 (7)	C20—C19—H19B	109.0
C11—C7—C8	109.07 (7)	C18—C19—H19B	109.0
C9—C8—C7	111.65 (7)	H19A—C19—H19B	107.8
C9—C8—H8A	109.3	C21—C20—C25	117.53 (9)
C7—C8—H8A	109.3	C21—C20—C19	119.93 (8)
C9—C8—H8B	109.3	C25—C20—C19	122.43 (8)
C7—C8—H8B	109.3	C22—C21—C20	122.15 (9)
H8A—C8—H8B	108.0	C22—C21—H21A	118.9
N1—C9—C8	111.00 (8)	C20—C21—H21A	118.9
N1—C9—H9A	109.4	C23—C22—C21	119.73 (9)
C8—C9—H9A	109.4	C23—C22—H22A	120.1
N1—C9—H9B	109.4	C21—C22—H22A	120.1
C8—C9—H9B	109.4	C22—C23—C24	119.32 (9)
H9A—C9—H9B	108.0	C22—C23—H23A	120.3
N1—C10—C11	110.70 (7)	C24—C23—H23A	120.3
N1—C10—H10A	109.5	C23—C24—C25	121.03 (9)
C11—C10—H10A	109.5	C23—C24—H24A	119.5
N1—C10—H10B	109.5	C25—C24—H24A	119.5
C11—C10—H10B	109.5	C24—C25—C20	120.20 (8)
H10A—C10—H10B	108.1	C24—C25—C26	117.20 (8)
C10—C11—C7	111.40 (8)	C20—C25—C26	122.60 (8)
C10—C11—H11A	109.3	O2—C26—O3	124.51 (9)
C7—C11—H11A	109.3	O2—C26—C25	119.16 (8)
C10—C11—H11B	109.3	O3—C26—C25	116.28 (8)
			
C6—C1—C2—C3	0.23 (16)	C12—C13—C14—C15	0.08 (19)
C1—C2—C3—C4	−0.97 (16)	C13—C14—C15—C16	−0.5 (2)
C1—C2—C3—Cl1	178.92 (8)	C14—C15—C16—C17	0.5 (2)
C2—C3—C4—C5	0.93 (17)	C15—C16—C17—C12	0.07 (19)
Cl1—C3—C4—C5	−178.95 (9)	C15—C16—C17—C18	−176.32 (12)
C3—C4—C5—C6	−0.16 (17)	C13—C12—C17—C16	−0.52 (17)
C4—C5—C6—C1	−0.55 (15)	C13—C12—C17—C18	175.89 (10)
C4—C5—C6—C7	178.51 (10)	C16—C17—C18—C19	103.89 (12)
C2—C1—C6—C5	0.52 (15)	C12—C17—C18—C19	−72.43 (12)
C2—C1—C6—C7	−178.55 (9)	C17—C18—C19—C20	−171.43 (8)
C5—C6—C7—O1	6.88 (12)	C18—C19—C20—C21	110.08 (11)
C1—C6—C7—O1	−174.08 (8)	C18—C19—C20—C25	−65.94 (12)
C5—C6—C7—C11	123.37 (10)	C25—C20—C21—C22	−1.84 (16)
C1—C6—C7—C11	−57.59 (11)	C19—C20—C21—C22	−178.06 (10)
C5—C6—C7—C8	−116.32 (10)	C20—C21—C22—C23	0.16 (17)
C1—C6—C7—C8	62.72 (11)	C21—C22—C23—C24	1.79 (16)
O1—C7—C8—C9	59.36 (10)	C22—C23—C24—C25	−2.03 (15)
C6—C7—C8—C9	−176.70 (8)	C23—C24—C25—C20	0.30 (14)
C11—C7—C8—C9	−55.42 (10)	C23—C24—C25—C26	−179.23 (9)
C10—N1—C9—C8	−55.59 (10)	C21—C20—C25—C24	1.59 (14)
C7—C8—C9—N1	55.39 (10)	C19—C20—C25—C24	177.71 (9)
C9—N1—C10—C11	55.93 (10)	C21—C20—C25—C26	−178.90 (9)
N1—C10—C11—C7	−56.19 (10)	C19—C20—C25—C26	−2.79 (14)
O1—C7—C11—C10	−62.75 (9)	C24—C25—C26—O2	125.98 (10)
C6—C7—C11—C10	176.40 (7)	C20—C25—C26—O2	−53.54 (13)
C8—C7—C11—C10	55.79 (10)	C24—C25—C26—O3	−51.43 (12)
C17—C12—C13—C14	0.45 (17)	C20—C25—C26—O3	129.05 (10)

### Hydrogen-bond geometry (Å, °)

**Table:**

Cg2 is the centroid of the C20–C25 ring.

**Table:**

*D*—H···*A*	*D*—H	H···*A*	*D*···*A*	*D*—H···*A*
O1—H1O1···O3	0.890 (18)	1.891 (18)	2.7401 (12)	158.8 (16)
N1—H1N1···O3^i^	0.954 (16)	1.754 (16)	2.6939 (11)	167.7 (15)
N1—H2N1···O2^ii^	0.917 (16)	1.818 (16)	2.7223 (11)	168.6 (15)
C8—H8B···Cg2	0.99	2.85	3.6743 (11)	141

Symmetry codes: (i) −*x*, *y*−1/2, −*z*+1/2; (ii) *x*, −*y*−1/2, *z*−1/2.

## References

[bb1] Bruker (2009). *APEX2*, *SAINT* and *SADABS* Bruker AXS Inc., Madison, Wisconsin, USA.

[bb2] Cosier, J. & Glazer, A. M. (1986). *J. Appl. Cryst.* **19**, 105–107.

[bb3] Cremer, D. & Pople, J. A. (1975). *J. Am. Chem. Soc.* **97**, 1354–1358.

[bb4] Cygler, M. & Ahmed, F. R. (1984). *Acta Cryst.* B**40**, 436–440.

[bb5] Cygler, M., Skarżyński, T., Skolimowski, J. & Thozet, A. (1980). *Acta Cryst.* B**36**, 2481–2483.

[bb6] Dutkiewicz, G., Siddaraju, B. P., Yathirajan, H. S., Siddegowda, M. S. & Kubicki, M. (2010). *Acta Cryst.* E**66**, o562. 10.1107/S1600536810004216 PMC2983646 21580330

[bb7] Jasinski, J. P., Butcher, R. J., Yathirajan, H. S., Mallesha, L. & Mohana, K. N. (2009). *Acta Cryst.* E**65**, o2365–o2366. 10.1107/S1600536809035363 PMC2970333 21577832

[bb8] Jasinski, J. P., Pek, A. E., Siddaraju, B. P., Yathirajan, H. S. & Narayana, B. (2010). *Acta Cryst.* E**66**, o2012–o2013. 10.1107/S1600536810026917 PMC3007433 21588324

[bb9] Sheldrick, G. M. (2008). *Acta Cryst.* A**64**, 112–122. 10.1107/S0108767307043930 18156677

[bb10] Spek, A. L. (2009). *Acta Cryst.* D**65**, 148–155. 10.1107/S090744490804362X PMC2631630 19171970

[bb11] Tomlin, D. W., Bunning, T. J., Price, G. E., Fratini, A. V. & Adams, W. W. (1996). *Acta Cryst.* C**52**, 1000–1002.

[bb12] Vartanyan, R. S. (1984). *Pharm. Chem. J.* **18**, 736–749.

